# Association of Renal Biochemical Parameters with Left Ventricular Diastolic Dysfunction in a Community-Based Elderly Population in China: A Cross-Sectional Study

**DOI:** 10.1371/journal.pone.0088638

**Published:** 2014-02-12

**Authors:** Jingmin Zhou, Xiaotong Cui, Xuejuan Jin, Jun Zhou, Hanying Zhang, Bixiao Tang, Michael Fu, Hans Herlitz, Jie Cui, Hongmin Zhu, Aijun Sun, Kai Hu, Junbo Ge

**Affiliations:** 1 Shanghai Institute of Cardiovascular Diseases, Zhongshan Hospital, Fudan University, Shanghai, China; 2 Fengjing Community Health Center, Jinshan District, Shanghai, China; 3 Section of Cardiology, Department of Medicine, Sahlgrenska University Hospital/Östra Hospital, University of Gothenburg, Gothenburg, Sweden; 4 Section of Nephrology, Department of Medicine, Sahlgrenska University Hospital/Östra Hospital, University of Gothenburg, Gothenburg, Sweden; University of São Paulo School of Medicine, Brazil

## Abstract

**Background:**

Relationship of left ventricular diastolic dysfunction (LVDD) with parameters that could provide more information than hemodynamic renal indexes has not been clarified. We aimed to explore the association of comprehensive renal parameters with LVDD in a community-based elderly population.

**Methods:**

1,166 community residents (aged ≥ 65 years, 694 females) participating in the Shanghai Heart Health Study with complete data of renal parameters were investigated. Echocardiography was used to evaluate diastolic function with conventional and tissue Doppler imaging techniques. Serum urea, creatinine, urea-to-creatinine ratio, estimated glomerular filtration rate (eGFR) and urinary albumin-to-creatinine ratio (UACR) were analyzed on their associations with LVDD.

**Results:**

The prevalence of LVDD increased in proportion to increasing serum urea, urea-to-creatinine ratio and UACR. These three renal parameters were found negatively correlated to peak early (E) to late (A) diastolic velocities ratio (E/A), and positively to left atrial volume index; UACR also positively correlated with E to peak early (E’) diastolic mitral annular velocity ratio (E/E’). Serum urea, urea-to-creatinine ratio and UACR correlated with LVDD in logistic univariate regression analysis, and urea-to-creatinine ratio remained independently correlated to LVDD [Odds ratio (OR) 2.82, 95% confidence interval (CI) 1.34–5.95] after adjustment. Serum urea (OR 1.18, 95%CI 1.03–1.34), creatinine (OR 6.53, 95%CI 1.70­–25.02), eGFR (OR 0.22, 95%CI 0.07–0.65) and UACR (OR 2.15, 95%CI 1.42–3.24) were revealed independent correlates of advanced (moderate and severe) LVDD.

**Conclusions:**

Biochemical parameters of renal function were closely linked with LVDD. This finding described new cardio-renal relationship in the elderly population.

## Introduction

Heart failure (HF) with preserved ejection fraction (HFPEF) has increasingly attracted attention for its high prevalence and poor prognosis [1]. Left ventricular diastolic dysfunction (LVDD) has been considered as one of the principal pathophysiologic mechanisms [2] and part of essential diagnostic indexes [3] of HFPEF. Both HFPEF and LVDD have been commonly demonstrated in aged population [4], [5]. Of note, the prevalence of chronic kidney disease (CKD) [6] and the CKD-associated mortality [7] were also significantly higher in HFPEF than HF with reduced ejection fraction (HFREF) among the elderly patients. However, the association between renal function and LVDD in elderly population has not been fully clarified. It was observed that renal insufficiency was associated with LVDD in patients with [8] or without [9] symptomatic HF. But so far, studies have mainly focused on the relationship between LVDD and dynamic renal parameters including estimated glomerular filtration rate (eGFR) and creatinine clearance [6]–[10]. However, renal biochemical parameters such as serum urea, urea-to-creatinine ratio and urinary albumin-to-creatinine ratio (UACR) that could provide additional information than dynamic renal indexes have not yet been reported in LVDD and HFPEF. Furthermore, most former studies were carried out in hospitalized patients while little is known about general population. In the present study, we aimed to explore the association of comprehensive renal parameters including serum urea, creatinine, urea-to-creatinine ratio, eGFR and UACR with LVDD in a community-based elderly population in China.

## Methods

### Ethics statement

The present study conforms to the ethical guidelines of the 1975 Declaration of Helsinki and was approved by the Ethics Committee of Zhongshan hospital affiliated to Fudan University. Written informed consent was obtained from each participant during recruitment.

### Study design and population

This study was part of Shanghai Heart Health Study (SHHS) [11], which is an ongoing large population study to investigate the incidence, prevalence, morbidity and mortality of cardiovascular diseases in community adults of Jinshan District of Shanghai, China. From January 2007 to June 2008, 1955 community residents older than 65 years were recruited and underwent a baseline survey including general information by self-administered questionnaire, standard 12-leads electrocardiogram (ECG), chest X-ray, and serum and urine biochemical parameters examination. The sample was followed up every 2 years. The biochemical parameters we used were measured in the first follow-up in 2009-2010. During the second follow-up in 2011, participants were invited to undergo an echocardiography examination. Finally 1166 participants with completed renal function and echocardiographic data were included in the present study. Those who did not have complete data from biochemical or echocardiographic analyses were excluded.

### Clinical, demographic and biochemical parameters

Clinical and demographic data were obtained from questionnaires at recruitment including age, gender, smoking and education status, and previous diseases and drug therapies. Blood pressure was measured with the manual mercury sphygmomanometer after a 5-min rest in a sitting position using phases 1 and 5 of the Korotkoff sounds for systolic and diastolic blood pressure (SBP and DBP), respectively. Height and body weight were measured simultaneously. Waist circumference was measured in midway between the lower limit of the rib cage and the iliac crest with the subject standing using a flexible and non-distensible tape. Heart rate (HR) was read out from standard 12-leads ECG measured after 5 minutes in supine position.

Coronary heart disease (CHD) was defined as a history of myocardial infarction or angina pectoris, or angiography confirmed coronary stenosis > 70%. Atrial fibrillation was documented by either medical history or the ECG examination, both paroxysmal and persistent atrial fibrillation were included. Stroke was diagnosed by past cranial computed tomography or magnetic resonance imaging. Those who had smoked more than three cigarettes a day for at least one year were defined as smokers, both former and current smokers were included. Diabetes mellitus was diagnosed by definite disease history, or using of oral hypoglycemic drugs or insulin, or fasting blood glucose (FBG) ≥ 7 mmol/l. Hypertension was diagnosed by persistent resting blood pressure greater than 140/90 mmHg, or a definite disease history. Body mass index (BMI) was calculated as weight (kg)/height (m)^2^. Obesity was a BMI of 28 kg/m^2^ or higher, or a waist circumference of at least 85 cm in men or 80 cm in women. Dyslipidemia was considered present if the subject had a definite disease history or met any of the following: total cholesterol (TC) ≥ 6.22 mmol/l, triglycerides ≥ 2.26 mmol/l, low-density lipoprotein cholesterol (LDL-C) ≥ 4.14 mmol/l, or high-density lipoprotein cholesterol (HDL-C) < 1.04 mmol/l.

Venous blood sample was obtained after a 12-hour-overnight fast and transferred every day to the central laboratory of Zhongshan hospital. After centrifugation and routine blood test, the serum was stored at −80°C until measurement of N-terminal pro-B type natriuretic peptide (NT-proBNP) using a fully automated electrochemiluminescence sandwich immunoassay on an Elecsys 1010 (Roche Diagnostics, Basel, Switzerland). Renal function parameters, and serum glucose and lipids were assessed by standard methods. The eGFR was calculated using the modified Modification of Diet in Renal Disease (MDRD) equations recommended by the Chinese eGFR Investigation Collaboration [eGFR (ml/min/1.73 m^2^)  = 175 × (Serum creatinine)^−1.234^× age^−0.179^ × 0.79 (if female) (Serum creatinine: mg/dl, age: years)] [12]. An overnight morning urine sample was also collected to measure the urinary albumin using the immune turbidimetric method (DiaSys Diagnostic Systems, Shanghai) and the urinary creatinine using the enzymatic colorimetric method (Roche/Hitachi). The UACR was calculated with these two parameters [UACR  =  urinary albumin × 1000/(urinary creatinine × 0.113)].

### Echocardiography

Echocardiograms were performed by using a commercially available echocardiography (Acuson Sequoia 218, Siemens, Germany) with a 2.5 or 3.5 MHz transducer according to the recommendations of the American Society of Echocardiography [13], [14] **.** Two-dimensional and Doppler images were obtained in the parasternal long and short axes and the apical 4- and 2-chamber long-axis views. M-mode echocardiograms of the left ventricle (LV) were recorded from the parasternal long-axis view. All recordings included at least 3 cardiac cycles and were digitally stored for off-line analysis. Left ventricular end-diastolic diameter (LVEDD), left ventricular end-systolic diameter (LVESD), interventricular septal thickness (IVST) and posterior wall thickness (PWT) were measured by M-mode or 2-dimensional echocardiography from the parasternal long axis view. Left ventricular mass (grams) was calculated as 0.80 × (1.04 × (IVST + LVEDD + PWT)^3^ − (LVEDD)^3^) + 0.6 according to Devereux et al [15], which was further divided by body surface area (BSA) as left ventricular mass index (LVMI). Left ventricular ejection fraction (LVEF) was calculated from LV end-systolic and end-diastolic volumes (LVESV and LVEDV) measured from the apical 4- and 2-chambers views, using the modified biplane Simpson’s method. Left atrial (LA) volume was measured with the same method, and was indexed to BSA as the LA volume index (LAVI). Doppler echocardiographic recordings were performed by pulsed wave Doppler with the sample volume at the tips of the mitral valve in the apical 4-chamber view. Peak early (E) and late (A) diastolic velocities, the E/A ratio, and the deceleration time (DT) of early diastolic velocity were measured as indicators of LV end-diastolic pressure. Assessment of peak early (E’) and late (A’) diastolic mitral annular velocity was performed by pulsed wave tissue Doppler imaging (TDI) of the lateral wall in the apical 4-chamber view.

### Criteria for diagnosis of LVDD

As recommended by the Heart Failure and Echocardiography Associations of European Society of Cardiology (ESC), both conventional and TDI echocardiographic techniques were used for diagnosis of LVDD [3]. In the present study, the LVDD diagnosis was made according to the criteria proposed by ESC [3] and to those adopted by Zhang et al in their study [16] with minor modifications. LVDD was considered to be present if any of the following criteria were met: (1) E/E’ > 8; (2) E/A < 0.5 and DT > 280ms; (3) LVMI > 149 g/m^2^ (male) or LVMI > 122 g/m^2^ (female); or (4) LAVI > 40 ml/m^2^.

### Classification of severity of LVDD

There are not uniformly recommended classifications for different degrees of LVDD. ESC guidelines in 2012 recommended 125 pg/ml of NT-proBNP as exclusion criteria of heart failure [5], while 220 pg/ml of NT-proBNP was previously shown to have positive predictive value of LVDD in diagnosis of HFPEF [3]. Therefore we chose 125 and 220 pg/ml as cut-off values to define mild LVDD with NT-proBNP < 125 pg/ml, moderate LVDD with NT-proBNP between 125 and 220 pg/ml, and severe LVDD with NT-proBNP ≥ 220 pg/ml, respectively.

### Statistical analysis

Participants were divided into two groups according to left ventricular diastolic function: normal diastolic function group and subjects with LVDD. Comparisons between the two groups were performed using *t* tests or Wilcoxon rank sum tests for continuous variables and chi-square tests for categorical variables. Comparisons of LVDD prevalence among tertiles of the renal parameter were analyzed by Pearson chi-square tests. Further pairwise comparisons used chi-square tests and Bonferroni method to judge the significance of differences. Considering that the parameters do not obey the bivariate normal distribution, we analyzed the relationship of renal parameters with echocardiographic indexes by Spearman correlation analysis and scatter plot. Logistic multivariate regression analysis was used to further investigate the relationship of renal parameters with LVDD and its severity. Variables adjusted in the models of logistic analysis included demographic variables (age, gender), clinical measures (BMI, heart rate, SBP), risk factors (hypertension, diabetes, dyslipidemia, smoking), cardiac co-morbidities (CHD, atrial fibrillation), biochemical parameters (FBG, NT-proBNP), and LVEF. All analyses were performed with Stata version 11.0 (College Station, TX, USA). *P* values less than 0.05 were considered to represent significance.

## Results

### Characteristics of participants

LVDD was diagnosed in 464 (39.8%) subjects out of the 1166 participants. Compared to participants without LVDD, subjects with it were more often female, more likely to have CHD, atrial fibrillation and hypertension, while less likely to smoke. Lower heart rate but higher levels of urea, urea-to-creatinine ratio, UACR and NT-proBNP were found in subjects with LVDD. Age, BMI, SBP, creatinine, eGFR and LVEF were similar between subjects with or without LVDD (**Table 1**).

**Table 1:** Characteristics of participants according to presence and absence of LVDD.

	Total	Without LVDD	With LVDD	*P* value*
**Demographics**				
n	1166	702	464	
Age (yrs)	72.7±4.8	72.7±4.7	72.9±4.9	0.658
Female (%)	59.5	55.7	65.3	0.001
Heart rate (bpm)	74±14	75±14	72±15	<0.001
BMI (kg/m^2^)	22.3±2.6	22.2±2.7	22.3±2.6	0.717
SBP (mmHg)	131±14	130±13	131±14	0.184
DBP (mmHg)	80±9	80±8	80±9	0.268
**Co-morbidities**				
CHD (%)	3.5	2.6	5.0	0.030
Atrial fibrillation (%)	4.6	2.1	8.4	<0.001
Stroke (%)	2.6	3.0	1.9	0.267
Hypertension (%)	44.4	41.7	48.5	0.023
Diabetes (%)	12.0	12.3	11.6	0.753
Dyslipidemia (%)	43.1	43.2	42.9	0.926
Smoking (%)	30.2	33.6	25.0	0.002
Obesity (%)	32.6	31.5	34.3	0.321
**Laboratory variables**				
Urea (mmol/l)	6.3±1.7	6.2±1.6	6.5±1.8	0.003
Creatinine (µmol/l)	64 (48–80)	64 (50–81)	62 (47–80)	0.098
Urea-to-creatinine ratio	96 (73–126)	92 (72–122)	102 (77–135)	<0.001
Uric acid (µmol/l)	339.8±94.9	338.7±94.5	341.5±95.5	0.762
eGFR (ml/min/1.73 m^2^)	108 (79–144)	106 (78–142)	111 (80–147)	0.320
UACR (µg/mg Cr)	14.0 (6.1–33.3)	12.8 (5.7­–30.6)	16.5 (7.0–39.2)	0.008
FBG (mmol/l)	5.9±1.5	5.9±1.6	5.9±1.4	0.500
TC (mmol/l)	4.9±1.0	4.9±1.0	4.9±1.0	0.479
TG (mmol/l)	1.7±1.0	1.7±1.1	1.6±1.0	0.440
LDL-C (mmol/l)	3.2±1.2	3.2±1.2	3.2±1.3	0.885
HDL-C (mmol/l)	1.4±0.4	1.4±0.4	1.4±0.3	0.884
NT-proBNP (pg/ml)	110.6 (65.2–193.7)	96.3 (57.8–173.6)	130.4 (77.1–232.8)	<0.001
LVEF (%)	64±9	65±8	64±10	0.660

Values of creatinine, urea-to-creatinine ratio, eGFR, UACR and NT-proBNP are median (interquartile), other values are mean ± SD or %.

HR, heart rate; BMI, body mass index; SBP, systolic blood pressure; DBP, diastolic blood pressure; CHD, coronary heart disease; eGFR, estimated glomerular filtration rate; UACR, urinary albumin-to-creatininie ratio; FBG, fasting blood glucose; TC, total cholesterol; TG, triglycerides; LDL-C, low density lipoprotein cholesterol; HDL-C, high density lipoprotein cholesterol; NT-proBNP, N-terminal pro-B type natriuretic peptide; LVEF, left ventricular ejection fraction.

*Compared between subjects with and without LVDD.

### Prevalence of LVDD in relation to different renal parameters

**Figure 1:**
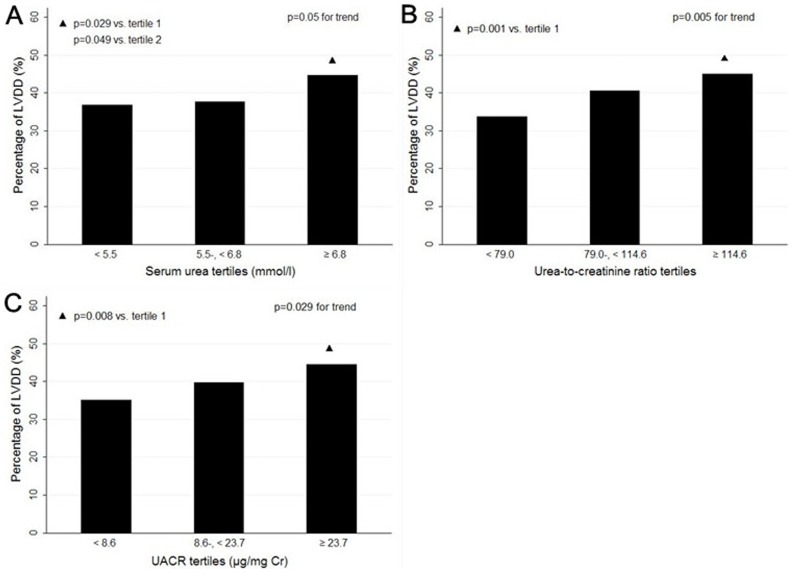
Prevalence of LVDD according to serum urea, urea-to-creatinine ratio, and UACR. The prevalence of LVDD is 36.9%, 37.6% and 44.6% in tertile 1, 2 and 3 of serum urea respectively. It is 33.8%, 40.5% and 45.1% for urea-to-creatinine ratio and 35.1%, 39.7% and 44.5% for UACR, respectively. LVDD, left ventricular diastolic dysfunction; UACR, urinary albumin-to-creatinine ratio.

The prevalence of LVDD increased in proportion to increasing values of serum urea (*P* = 0.05), urea-to-creatinine ratio (*P* = 0.005) and UACR (*P* = 0.029) (**Figure 1**). The prevalence of LVDD was significantly higher in subjects with serum urea (*P* = 0.029), urea-to-creatinine ratio (*P* = 0.001) and UACR (*P* = 0.008) located in the top tertile than in the bottom. This trend was not observed with respect to creatinine and eGFR (Data not shown).

### Relationship of renal parameters with echocardiographic indexes

**Figure 2:**
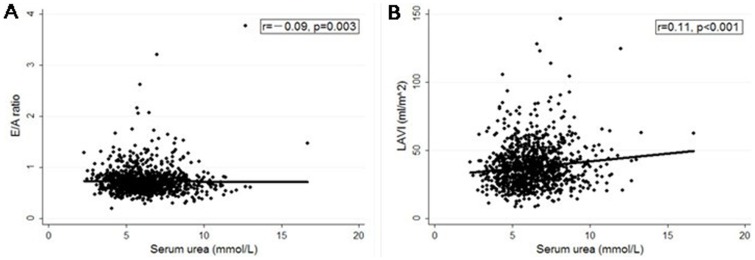
Relationship between serum urea and echocardiographic indexes (E/A and LAVI). **A)** Serum urea was negatively correlated to E/A ratio. **B**) Serum urea was positively correlated to LAVI. LAVI, left atrial volume index.

**Figure 3:**
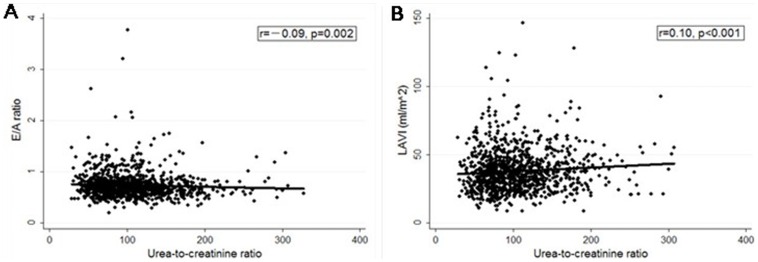
Relationship between urea-to-creatinine ratio and echocardiographic indexes (E/A and LAVI). **A**) Urea-to-creatinine ratio was negatively correlated to E/A ratio. **B**) Urea-to-creatinine ratio was positively correlated to LAVI. LAVI, left atrial volume index.

**Figure 4:**
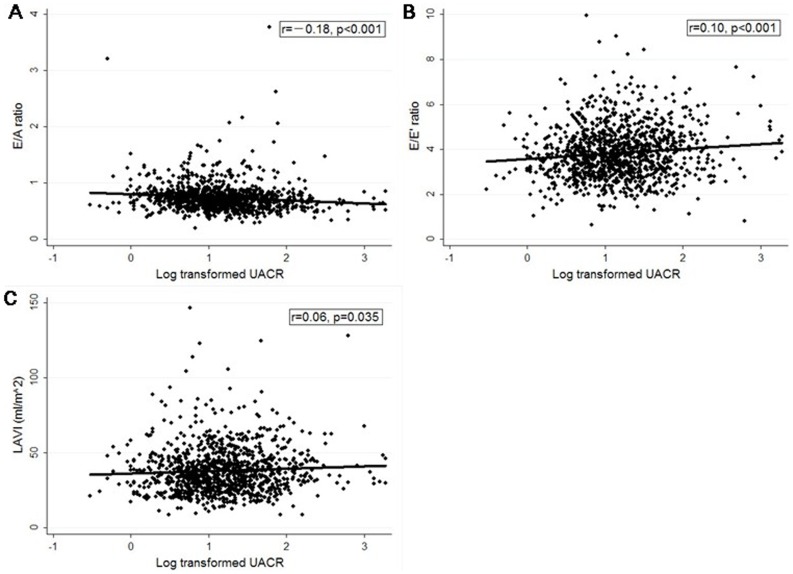
Relationship between UACR and echocardiographic indexes (E/A, E/E’ and LAVI). **A)** UACR was negatively correlated to E/A ratio. **B**) UACR was positively correlated to LAVI. **C**) UACR was also positively correlated to E/E’ ratio. UACR, urinary albumin-to-creatinine ratio; LAVI, left atrial volume index.

Serum urea (**Figure 2**), urea-to-creatinine ratio (**Figure 3**) and UACR (**Figure 4**) were negatively correlated to E/A ratio and positively correlated to LAVI. Besides, UACR was positively correlated to E/E’ ratio (**Figure 4**).

### Association of renal parameters with presence and severity of LVDD

Logistic univariate analysis showed that serum urea [odds ratio (OR) 1.12, 95% confidence interval (CI) 1.04–1.20], urea-to-creatinine ratio (OR 3.02, 95% CI 1.56–5.86) and UACR (OR 1.30, 95% CI 1.05–1.60) were associated with LVDD in this cohort. After adjusted for confounders, urea-to-creatinine ratio remained as an independent correlate of LVDD (**Table 2**). Neither serum creatinine nor eGFR correlated with LVDD before or after adjustment. **Table 2** also showed that serum urea, creatinine, eGFR and UACR were all independently correlated to the advanced LVDD in the multivariate logistic analysis. Parallely, these parameters were correlated to NT-proBNP level (**Table 3**).

**Table 2:** The association of biochemical renal parameters with LVDD and severity of LVDD.

Variable***	LVDD*			Advanced LVDD**		
	OR	95% CI	*P* value	OR	95% CI	*P* value
Urea (mmol/l)	1.07	0.99–1.16	0.087	1.18	1.03–1.34	0.013
Creatinine (µmol/l)	0.47	0.21–1.06	0.070	6.53	1.70–25.02	0.006
Urea-to-creatinine ratio	2.82	1.34–5.95	0.006	0.58	0.18–1.94	0.379
eGFR (ml/min/1.73 m^2^)	1.85	0.95–3.60	0.070	0.22	0.07–0.65	0.006
UACR (µg/mg Cr)	1.10	0.86–1.40	0.451	2.15	1.42–3.24	<0.001

OR, odds ratio; CI, confidence interval. Other abbreviations as in Table 1.

*Adjusted for age, gender, heart rate, BMI, SBP, smoking status, presence or absence of hypertension, diabetes mellitus, CHD, and atrial fibrillation; FBG, NT-proBNP and LVEF. NT-proBNP is log transformed before entering models.

**Adjusted the same parameters except NT-proBNP.

***Per 1 unit increase in urea values; per 1 log unit increase in creatinine, urea-to-creatinine ratio, eGFR and UACR values.

**Table 3:** The association of biochemical renal parameters with NT-proBNP.

Variable	NT-proBNP	
	*r* *	*P* value
Urea (mmol/l)	0.12	<0.001
Creatinine (µmol/l)	0.07	0.021
Urea-to-creatinine ratio	0.01	0.667
eGFR (ml/min/1.73 m^2^)	–0.13	<0.001
UACR (µg/mg Cr)	0.18	<0.001

Abbreviations as in Table 1.

*r, Spearman correlation coefficient.

### Subgroup analysis in participants with normal renal function

Most participants in community have normal renal function. So further analysis was made in a subgroup with eGFR > 60 ml/min/1.73 m^2^. In this subgroup, urea-to-creatinine ratio was still independently correlated to LVDD. While creatinine was negatively and eGFR was positively correlated to LVDD (**Table 4**).

**Table 4:** The association of renal parameters with LVDD in subpopulation with eGFR > 60 ml/min/1.73 m^2^.

Variable	Unadjusted			Adjusted*		
	OR**	95% CI	*P* value	OR**	95% CI	*P* value
Urea (mmol/l)	1.10	1.01-1.19	0.021	1.07	0.98-1.16	0.138
Creatinine (µmol/l)	0.28	0.11-0.69	0.006	0.33	0.12-0.90	0.031
Urea-to-creatinine ratio	3.93	1.89-8.15	<0.001	3.06	1.35-6.92	0.007
eGFR (ml/min/1.73 m^2^)	2.18	1.00–4.72	0.049	2.48	1.09–5.64	0.031
UACR (µg/mg Cr)	1.26	1.01–1.59	0.045	1.10	0.84–1.43	0.491

OR, odds ratio; CI, confidence interval.

*Adjusted for age, gender, heart rate, BMI, SBP, smoking status, presence or absence of hypertension, diabetes mellitus, CHD, and atrial fibrillation; FBG, NT-proBNP and LVEF. NT-proBNP is log transformed before entering models. Abbreviations as in Table 1.

**OR is per 1 unit increase in urea values; per 1 log unit increase in creatinine, urea-to-creatinine ratio, eGFR and UACR values.

## Discussion

In the present study, we found for the first time in our knowledge that higher levels of serum urea, urea-to-creatinine ratio and UACR were correlated to increasing prevalence of LVDD in a community-based elderly population of China, and this was supported by their negative correlations to E/A ratio and positive correlations to LAVI and E/E’. Moreover, urea-to-creatinine ratio was an independent correlate of LVDD. Thus, renal function has proved to be closely linked with LVDD in this community-based elderly population.

Although serum urea has been demonstrated to be predictive of adverse outcome in HFPEF patients [17], [18], to the best of our knowledge, its relationship with LVDD in conjunction with other measures of renal function has not been investigated in the general elderly population. Traditionally, serum urea has been considered as a less specific marker of renal function than GFR, since it is affected by extra-renal factors like protein intake and catabolism [19]. However, serum urea may rise independently of changes in GFR because of enhanced proximal and distal tubular reabsorption by the effect of arginine-vasopressin (AVP) on the urea transporter under neurohormonal activation [19**]**–[21]. Recently, McKie et al reported that patients with preclinical diastolic dysfunction showed impaired renal cyclic guanosine monophosphate (cGMP) activation which contributed to impaired natriuresis in response to volume expansion [22]. The impaired natriuresis indicates higher reabsorption of urea and increasing serum urea concentration. These previous findings about the cardio-renal response suggest a possible explanation for the association between higher urea levels and LVDD observed in the present study. Furthermore, in the work by McKie et al [22], impaired cGMP activation and natriuresis were not paralleled by increased E/E’, which is consistent with our finding that serum urea was related with E/A and LAVI but not E/E’.

The serum urea-to-creatinine ratio has been extensively used for the differentiation of “pre-renal” dysfunction from intrinsic renal parenchymal disease [23], and has been suggested positively correlated with poor outcomes in patients with acute or chronic HF [24**]**–[26]. Neuroendocrine activation might also serve as one of the potential reasons for urea-to-creatinine ratio elevation in pre-renal ischemia [20], [21]. Therefore, the aforementioned mechanism with regard to urea and LVDD could also interpret the independent relation of urea-to-creatinine ratio with LVDD. Our findings about a positive relation of urea and urea-to-creatinine ratio with LVDD but lack of relation of creatinine and eGFR with LVDD may suggest a neuroendocrine activation status in LVDD subjects. We hypothesize that serum urea and urea-to-creatinine ratio are more sensitive than eGFR and creatinine to represent the early neuroendocrine activation in LVDD, when the cardiac output has not reduced yet and glomerular filtration is still normal. Further studies are warranted to test these relationships and the hypothesis.

Available findings about urinary albumin excretion (UAE) and its relation with diastolic function remain conflicting [27**]**–[30]. The CHARM study found that the prevalence of increased UACR, a convenient method for detection of UAE [27], was similar in HFPEF (41%) and HFREF patients [28], whereas HARVEST study reported that UAE was unrelated to LV mass in subjects with stage 1 hypertension [29] **.** Furthermore, Post et al found that elevated random spot UACR was a marker of increased LV mass [30] **.** In the present study, we found 1) the prevalence of LVDD increased with the increasing UACR, 2) UACR was related to echocardiographic indexes like E/E’, E/A and LAVI, and 3) UACR was independently associated with the severity of LVDD. Therefore our findings not only extend previous studies by Post et al but also suggest LVDD as pathophysiologic link between UAE and mortality [31]. Endothelial dysfunction might represent a contributory mechanism to this association since it has been proved to be associated with both UAE [32], [33] and LVDD [34]–[36].

UACR is suggested not specific for kidney disease but a marker of systemic inflammation and microvascular damage including endothelial dysfunction [37], [38] **,** which is an predominant early step in the process of clinical overt cardiovascular disease [39] **.** Nevertheless, eGFR mainly reflects the hemodynamic status of the kidney**.** Since isolated LVDD was found associated with endothelial dysfunction [34]–[36] but unlikely to cause decline of cardiac output and renal macrovascular perfusion in the early stage, it could be understood why LVDD was correlated with UACR but not with eGFR in the present study.

Both population-based studies [40], [41] and studies on hospitalized HFPEF patients [42], [43] support our choice of NT-proBNP as tool to grade LVDD since in both settings NT-proBNP was found to be directly associated with LVDD severity. The relationship of renal parameters with NT-proBNP uncovered in our study, together with other studies demonstrating that NT-proBNP levels are affected by declining renal function [44**]**–[46], are in keeping with the theory that the clearance of NT-proBNP occurs solely in the kidney [47]. This finding also indicates that when NT-proBNP is employed to evaluate the presence or severity of LVDD, renal function should be considered because both decreased clearance of kidney and increased production due to LVDD could lead to the rise of NT-proBNP.

The subgroup analysis in subjects with normal renal function (eGFR > 60 ml/min/1.73 m^2^) revealed, for the first time, an interesting result that eGFR was independently and positively correlated to LVDD. It has been demonstrated that there is a significant and positive correlation between GFR and LV mass in non-diabetic patients with mild to moderate hypertension [48], concurring with our result to some extent. Considering these findings, we assume that glomerular hyperfiltration may be linked to LVDD. Nevertheless, more studies are still needed to confirm this assumption.

The present study has some limitations. First, specific data on symptoms or signs related to HF were not available. Additionally, we did not assess Ar_d_ **–**A_d_ because of constraints in echocardiographic examination time per participant. Ar_d_ **–**A_d_ was still kept in the latest European Consensus document as a second-line criteria of LVDD [3]. However, studies suggested that Ar_d_ **–**A_d_ was often obtained less successfully (49%**–**84%) in clinic than other diastolic measures [49], [50], and pulmonary venous inflow had more inter-reader variability than mitral valve inflow and annular tissue Doppler [50]. Emery et al also found that even in hospitalized patients, very few of them presented Ar_d_ **–**A_d_ > 30ms, and the measure had minimal sensitivity [49]. All of these indicated that Ar_d_ **–**A_d_ probably contribute little to overall diastolic function assessment.

## Conclusions

In summary, the present study suggests that higher levels of serum urea, urea-to-creatinine ratio and UACR are linked with increasing prevalence of LVDD, and urea-to-creatinine ratio is an independent correlate of LVDD. Although serum creatinine and eGFR are not associated with LVDD in the whole community cohort, they, together with serum urea and UACR, are independently associated with advanced LVDD in parallel with increased NT-proBNP level. Our findings contribute to further understanding of the cardio-renal relationship in the elderly population.
